# Fried food intake and risk of nonfatal acute myocardial infarction in the Costa Rica Heart Study

**DOI:** 10.1371/journal.pone.0192960

**Published:** 2018-02-15

**Authors:** Peter Hu, Yanping Li, Hannia Campos

**Affiliations:** 1 Department of Nutrition, Harvard T.H. Chan School of Public Health, Boston, Massachusetts, United States of America; 2 Cornell University College of Human Ecology, Ithaca, New York, United States of America; 3 Centro de Investigación e Innovación en Nutrición Traslacional y Salud (CIINT), Universidad Hispanoameriana, San Jose, Costa Rica; Universitat de Lleida-IRBLLEIDA, SPAIN

## Abstract

Economic development in middle-income countries has led to a noticeable rise in the availability of commercial deep fried foods and lifestyles that require eating meals “on the go” and outside of the home. Yet, data from these countries where fried foods were traditionally prepared at home are scarce, despite several studies showing the potential adverse effects of fried food consumption on risk for heart disease. We aimed to examine whether consumption of fried foods inside or outside of the home is associated with an increased risk of myocardial infarction (MI) among Hispanic/Latinos living in Costa Rica. Participants were incident cases of a first acute MI (n = 2,154) and randomly selected controls matched for age, sex, and residence (n = 2,154). After adjustment for traditional cardiovascular risk factors, including history of diabetes, history of hypertension, smoking, abdominal obesity, income, educational years, occupation, alcohol intake, dietary intakes of saturated fatty acid, fiber intake, and total energy intake, the multivariable-adjusted odds ratio (OR, 95% CI) for risk of MI were 1.00 (reference), 1.02 (0.86–1.21), 1.26 (0.81–1.95), and 1.58 (1.08–2.30) for intake of fried foods outside of the home <1/week, 1-3/week, 4-6/week, and 1/day, respectively (*P* trend = 0.02); and 1.00, 0.81 (0.65–1.00), 0.81 (0.61–1.09), and 0.93 (0.72–1.19), respectively (*P* for trend = 0.65) for intake of fried foods inside the home. The data suggest that consumption of fried foods outside of the home, a practice that has been associated with economic development, could have adverse effects on cardiovascular disease.

## Introduction

Historically, food preparation and consumption in low- and middle-income countries has been restricted to the home. Costa Rican families prepare meals by various means, including frying, a method in which food is submerged in hot oil [1]. More recently, economic development in low- and middle-income countries has resulted in changes to food preparation and consumption, including higher intake of refined grains, sodium, and red meat [2,3]. These foods are characteristic of being highly processed and packaged for “on the go” eating or eating outside of the home. In fact, the entire food system, lifestyles, cooking methods, and eating practices are changing rapidly [1,2]. For example, in China, the energy contributions from snacks, foods eaten outside of home, and foods prepared outside of home have increased over time [4]. In Latin America, retail food sales increased from 15% in 1990 to 60% by 2000 in Latin American supermarkets [5]. While frying is not a new technique in Costa Rica, people are currently eating more fried foods and foods outside the home compared to 30 years ago [6]. Higher consumption of fried food outside the home, resulting from modernization, economic development and urbanization, could be associated with increased risk of cardiovascular disease CVD.

Although frying improves taste and aroma, fried foods may have detrimental cardiovascular effects [7]. A cross-sectional study in Spain found that food fried with re-used oils was associated with a higher prevalence of arterial hypertension [8]. Fried food intake was also associated with higher body weight and risk of obesity [9,10] and type 2 diabetes among US men [11] and women [11,12]. Italian adults with a higher intake of fried food had lower high density lipoprotein cholesterol levels and larger waist circumferences [13]. A positive association between fried foods and risk of myocardial infarction (MI) was observed in INTERHEART, a case-control study of 52 countries [14]. In contrast, the Spanish cohort of the European Prospective Investigation into Cancer and Nutrition (EPIC) study found no association between fried food and risk of coronary heart disease [15]. The authors concluded that the type of frying oil was the main reason for the differences between studies: olive and sunflower oils are the most commonly used fats for frying in Spain and are less prone to oxidation than other edible oils or fats [16].

Interestingly, results from two large, prospective cohorts that evaluated consumption of fried foods inside versus outside the home showed that fried food intake was significantly associated with risk of incident type 2 diabetes and coronary artery disease, primarily when the fried foods were eaten outside of the home [11]. These studies suggest that unique characteristics of foods fried away from home such as the type and amount of oil used, portion size, and the carbohydrate quality of the food being fried may be responsible for their potential adverse effects [17]. Our previous study in Costa Rica found no association between consumption of fried foods and risk of nonfatal acute MI, but we did not distinguish between eating fried foods inside and outside of the home [1]. Since fried foods eaten away from home may have a unique impact on risk of MI [11], the purpose of the present study was to examine whether intake of fried foods inside and outside the home is associated with the risk of MI in the Costa Rica Heart Study.

## Methods

### Study population and design

The study design and population of the Costa Rica study has been described previously [1,18]. In summary, all subjects were Hispanic Americans of Mestizo background who lived in the Central Valley of Costa Rica. Eligible cases were men and women who were determined to be survivors of a first acute MI by 2 independent cardiologists at any of the 6 recruiting hospitals in the catchment area. To achieve 100% ascertainment, fieldworkers visited the 6 hospitals daily. All cases met the World Health Organization criteria for MI, which require typical symptoms plus either elevations in cardiac biomarker concentrations or diagnostic changes on an electrocardiogram [19]. Cases were ineligible if they 1) died during hospitalization, 2) were 75 y or older on the day of their first MI, or 3) were physically or mentally unable to answer the questionnaire. Enrollment was carried out while the cases were in the hospital’s step-down unit. Cases were matched by age (±5 y), sex, and area of residence to population control subjects who were randomly identified with data from the National Census and Statistics Bureau of Costa Rica. Because of the comprehensive social services provided in Costa Rica, all persons living in the catchment area had access to medical care regardless of income. Therefore, the control subjects came from the source population that gave rise to the cases and were not likely to have had undiagnosed cardiovascular disease because of poor access to medical care. Control subjects were ineligible if they ever experience a MI or if they were physically or mentally unable to complete the questionnaires. After enrollment of cases at the hospital step-down unit, all cases and controls were visited at their homes for the collection of dietary and health information, anthropometric measurements, and biological specimens [1,18]. All subjects gave informed consent on documents approved by the Human Subjects Committee of the Harvard School of Public Health and the University of Costa Rica. To avoid the potential for recall bias among the cases, data were collected as close to the diagnosis of MI as possible.

### Data collection

Socio-demographic characteristics, smoking status, socioeconomic status, physical activity, and medical history data were collected at the in-home interview. Dietary data was collected by using a semi-quantitative food-frequency questionnaire (FFQ) that was developed and validated specifically to assess nutrient intake in the Costa Rican population [20]. In addition to foods, the FFQ inquired about the frequency of eating fried foods outside of the home as well as inside the home: <1 times/week or never, 1–3 times/week, 4–6 times/week, or daily.

### Statistical analysis

The original population consisted of 2,274 case-control pairs and 27% of them were women. Participants with missing values for fried food consumption inside or outside the home were deleted (n = 123); if a case or control was missing, the whole pair was deleted. If among the deleted controls a participant that had complete data matched a deleted case by age, sex, and area of residence, the two participants were rematched and added to the data set. Thus, 3 cases and 3 rematched controls with complete data were included in the final data set for a total of 2,154 case-control pairs. The present study was based on the fixed sample size of the 2,154 case-control pairs.

Because of the matched design, the significance of differences in the distributions of categorical variables by case-control status and frequency of eating fried foods outside of the home was tested using McNemar’s test. If normally distributed, continuous variables were tested by the paired t test; otherwise, the Wilcoxon signed rank test was used. Differences were considered significant at *P*< 0.05. To quantify a linear trend, we conducted a Wald test for linear trends by assigning the median value to each category of frequency of eating fried foods outside of the home and modeling this variable as a continuous variable. We applied the conditional logistic regression model to estimate the odds ratios (OR) and 95% confidence intervals (CI) for MI comparing participants with different frequencies of eating fried foods inside or outside of home, with participants who ate fried food inside or outside of the home <once per week as the reference group. In our multivariable analyses, we adjusted for established cardiovascular risk factors: history of diabetes (yes/no), hypertension (yes/no), smoking (never, past, <10 cigarettes/d, 10–19 cigarettes/d, and ≥20 cigarettes/d), waist-hip-ratio (quintiles), physical activity (quintiles), income (quintiles), educational duration (years), occupation (retired, agriculture, plumbers, semi-skilled or driver, managers and administrators, professionals and others), and intake of alcohol (never, past, and tertile of alcohol intake among current drinkers).

To examine the extent to which dietary factors explained the association between frequency of eating fried foods inside or outside of the home and risk of MI, we estimated the magnitude of change in the regression coefficient for eating fried foods inside or outside of the home with and without adjustment for each individual potential mediator, including dietary energy intake, dietary fiber intake, and energy contribution from saturated fat. The association between the percentage of eating fried foods outside of home and MI was explained by the dietary intermediate variables was computed as follows: (1 –(βmediator-adjusted model / βmultivariable model)) X 100% [21]. A positive change in the regression coefficient indicates a change in the rate ratio towards the null. SAS macro %MEDIATE was applied (publicly available at www.hsph.harvard.edu/faculty/spiegelman/mediate.html) [21].

To address the possibility of residual confounding, we applied the propensity score method in the sensitivity analysis. We estimated the probability of fried food intake conditional on observed covariates [22], using frequencies of fried food intake as a dependent variable and all the covariates listed above for model 4 as independent variables. The propensity scores of different frequencies of fried food intake were included in the analysis as continuous variables and the effect of fried food intake on outcomes was estimated based on the adjustment of these propensity scores [22,23].

In the sensitivity analysis, we also further adjusted for the type of cooking oil and simultaneously adjusted for the frequency of eating fried foods inside and outside of the home. In order to maximally control the residual confounding from other dietary intakes, we also did another sensitivity analysis: instead of adjustment for individual food and nutrient items, we adjusted for the index of factors of the dietary pattern identified by principal component analysis, which had been found to be associated with risk of MI in our study [24]. We also performed subgroup analyses to explore the effects of eating fried foods outside of the home stratified by several covariates. In these stratification analyses, because the matched pairs might be separated into different subgroups, we used unconditional logistic regression with matching variables and other potential confounders in the model for each subgroup analysis. In all unconditional analyses, we computed the Hosmer-Lemeshow statistic to test for the goodness of-fit of the models. We examined potential interactions of eating fried foods outside of the home with the stratifying variables (smoking, physical activity, sex, and obesity) on risk of MI by including a multiplicative term in the model with adjustment for other potential confounders. We applied the conditional logistic regression model in the test of interactions, which were based on the whole study population.

SAS software version 9.4 (SAS Institute Inc, Cary, NC) was used for all statistical analyses, and all *P* values presented are two-tailed.

## Results

### General characteristics

The characteristics of the cases and population-based matched controls in Costa Rica are shown in Table 1. Compared to controls, the cases were more likely to have abdominal obesity, history of diabetes and hypertension, lower physical activity, and less income. Cases had fewer current drinkers but were more likely to smoke and have a less formal education, and were more likely to consume a diet high in total energy and saturated fat, but low in polyunsaturated fat and fiber.

**Table 1:** Characteristics of nonfatal MI cases and population-based matched control in the Costa Rica Heart Study^1^.

Variable	Controls	Cases	P*
Age (y)^2^	58.2(11.3)	58.5(11.0)	N/A
Women (%)^2^	27	27	N/A
Living in Rural Area (%)^2^	26	26	N/A
History of Diabetes (%)	14	25	< .0001
History of Hypertension (%)	29	39	< .0001
Current smoker (%)	21	40	< .0001
Current alcohol drinker (%)	53	48	0.004
Alcohol intake among drinkers (g/day)^3^	11.2(17.1)	13.2(23.9)	0.02
Waist circumference (cm)	90.9(10.0)	90.8(9.3)	0.95
Physical activity (METs)^4^	35.5(16.2)	34.3(16.2)	0.02
Formal education (y)	7.5(5.3)	7.1(5.4)	0.009
Occupation (%)			0.15
Retired	18.7	19.5	
Agriculture	6.7	6.6	
Plumbers, semi-skilled, driver	24.3	27.1	
Managers and administrators	20.9	18.2	
Professionals	26.7	26.2	
Students or others	2.7	2.6	
Monthly household income (US$)	569(426)	496(392)	< .0001
Dietary intake			
Energy (kcal)	2443(765)	2703(947)	< .0001
Saturated fat (% of energy)	11.7(2.9)	12.4(3.1)	< .0001
Polyunsaturated fat (% of energy)	7.1(2.3)	6.8(2.3)	0.0005
*Trans* fat (% of energy)	1.31(0.64)	1.33(0.64)	0.30
Cholesterol (mg/1000 kcal)	118(52)	126(58)	< .0001
Carbohydrate (% of energy)	55.4(7.3)	54.4(7.5)	< .0001
Protein (% of energy)	12.9(2.1)	13.2(2.2)	0.0004
Fiber (g/d)^3^	25.1(6.1)	24.1(6.4)	< .0001
Alpha linolenic acid (g/d)^3^	1.59(0.77)	1.69(0.85)	< .0001
Eating fried foods at home			<0.0001
<1 time/week	12.9	13.8	
1–3 times/week	53.4	47.2	
4–6 times/week	10.8	9.8	
daily	22.9	29.2	
Eating fried food outside the home			<0.0001
< 1 time/week	69.9	65.5	
1–3 times/week	25.4	25.4	
4–6 times/week	2.1	3.3	
daily	2.9	5.8	

^1^Values are means (SD) or %.

* Significance for different between cases and controls (McNe-mar’s or paired t test or Wilcoxon signed rank test P <0.05).

^2^Matching variable

^3^Adjusted for total energy intake using the residual method

^4^METs, Metabolic Equivalent of Tasks

The general characteristics and potential confounders among population controls are shown in Table 2.

**Table 2:** Characteristics of population-based matched controls by frequency of fried food intake at home and outside of the home^1^.

	<1 time/week	1–3 times/week	4–6 times/week	Daily	*P* for trend
Frequency of eating fried foods inside home					
N	277	1,150	233	494	
Women (%)^2^	43	26	24	21	< .0001
Living in urban area (%)^2^	75	73	78	74	0.56
Age (years)^2^	61(11)	58(11)	57(11)	57(12)	0.0004
Waist Circumference (cm)	89.8(9.5)	90.8(10.2)	91.3(10.4)	91.3(9.8)	0.09
Physical Activity (METs)^3^	33.1(13.0)	35.1(15.5)	36.3(14.7)	37.3(19.3)	0.0003
Current smoker (%)	20	21	19	23	0.45
Current alcohol drinker (%)	46	53	53	55	0.12
Monthly household income (US$)	557(456)	571(436)	620(411)	547(392)	0.74
Education (years completed)	7.09(5.45)	7.55(5.34)	8.31(5.49)	7.42(5.10)	0.46
History of diabetes (%)	16	11	13	16	0.05
History of hypertension (%)	34	30	27	25	0.006
Total energy (kcal)	2029(663)	2378(695)	2544(743)	2781(837)	< .0001
Saturated fat (% of energy)	10.7(3.1)	11.5(2.8)	12.2(2.9)	12.5(2.9)	< .0001
Polyunsaturated fat (% of energy)	6.9(2.4)	7.0(2.3)	7.0(2.3)	7.3(2.4)	0.004
*Trans* Fat (% of energy)	1.17(0.61)	1.34(0.67)	1.29(0.58)	1.36(0.62)	0.04
Alpha linolenic acid (g/d)^4^	1.33(0.66)	1.51(0.71)	1.66(0.71)	1.88(0.89)	< .0001
Cholesterol (mg/1000 kcal)	97(48)	114(45)	118(42)	139(64)	< .0001
Carbohydrate (% of energy)	57.4(8.5)	55.8(7.1)	54.6(6.9)	53.8(6.9)	< .0001
Protein (% of energy)	12.8(2.6)	12.9(2.0)	12.9(2.0)	13.1(2.0)	0.03
Fiber (g/d)^4^	25.7(6.5)	25.2(5.7)	24.4(6.0)	24.6(6.6)	0.006
Type of cooking oil in home (%)					< .0001
Sunflower	23	24	17	17	
Palm	19	22	26	27	
High trans soybean oil	18	25	16	19	
Low trans soybean oil	32	26	36	35	
Others	8	3	5	2	
Occupation (%)					0.002
Retired	19	19	19	17	
Agriculture	4	7	7	8	
Plumbers, semi-skilled, driver	18	25	22	28	
Managers and administrators	18	22	19	21	
Professionals	38	25	27	23	
Others	3	2	5	2	
Frequency of eating fried foods outside of the home					
N	1,499	546	46	63	
Women (%)^2^	30	20	11	8	< .0001
Living in urban area (%)^2^	73	77	70	86	0.03
Age (years)^2^	60(11)	54(12)	52(12)	51(11)	< .0001
Waist Circumference (cm)	90.3(10.0)	92.0(10.0)	93.2(9.5)	91.8(9.8)	0.005
Physical Activity (METs)^3^	35.4(16.1)	35.8(16.7)	33.9(15.4)	36.0(12.5)	0.85
Current smoker (%)	20	23	30	27	0.14
Current alcohol drinker (%)	48	62	70	65	< .0001
Monthly household income (US$)	528(410)	656(446)	793(479)	610(437)	< .0001
Education (years completed)	7.05(5.18)	8.69(5.51)	8.72(5.44)	8.44(5.24)	< .0001
History of diabetes (%)	16	10	13	16	0.03
History of hypertension (%)	32	25	28	16	0.002
Total energy (kcal)	2323(695)	2630(766)	3062(920)	3222(1157)	< .0001
Saturated fat (% of energy)	11.3(3.0)	12.3(2.6)	13.4(2.9)	13.8(2.6)	< .0001
Polyunsaturated fat (% of energy)	7.0(2.4)	7.2(2.1)	6.8(2.4)	7.5(2.3)	0.14
*Trans* Fat (% of energy)	1.30(0.65)	1.34(0.63)	1.30(0.47)	1.34(0.59)	0.52
Alpha linolenic acid (g/d)^4^	1.53(0.75)	1.69(0.76)	1.70(0.74)	1.96(0.94)	< .0001
Cholesterol (mg/1000 kcal)	117(54)	118(44)	129(67)	125(48)	0.09
Carbohydrate (% of energy)	56.4(7.2)	53.5(7.1)	52.1(7.0)	50.5(5.6)	< .0001
Protein (% of energy)	12.9(2.1)	13.0(2.0)	12.2(1.8)	13.0(2.0)	0.55
Fiber (g/d)^4^	25.5(5.7)	24.3(6.6)	21.3(5.6)	22.8(6.6)	< .0001
Type of cooking oil in home (%)					0.86
Sunflower	21	22	24	22	
Palm	24	21	28	27	
High trans soybean oil	21	23	22	22	
Low trans soybean oil	30	30	24	29	
Others	4	4	2	0	
Occupation (%)					< .0001
Retired	23	11	2	5	
Agriculture	7	5	4	3	
Plumbers, semi-skilled, driver etcs	23	24	48	40	
Managers and administrators	16	32	24	40	
Professionals	28	25	20	11	
Others	3	3	2	2	

^1^Values are means (SD) or % (percentage of population in each category);

^2^Matching variable;

^3^MET, metabolic equivalent of task

^4^Adjusted for total energy intake using the residual method and does not include supplements.

Compared to those who ate fried foods less than once per week inside or outside the home, controls who ate fried food daily either inside or outside the home were younger, less likely to be women, less likely to report hypertension, and had higher dietary intakes of total energy, saturated fat and ALA. Controls who ate fried foods daily also had less intakes of dietary fiber and carbohydrates, and were more likely to use soybean and sunflower oil than palm oil (Table 2). Compared to controls who seldom ate fried food at home, controls who ate fried foods daily at home were more physically active and had higher intakes of polyunsaturated fat, trans fat, cholesterol and protein. Controls with frequent fried food consumption outside of the home had lower family incomes, lower educational levels, and were more likely to drink alcohol compared to controls who ate fried food outside of the home less than once per week. The proportion of plumbers, semi-skilled workers, and drivers was higher among controls who ate fried food daily outside the home than the proportion among controls who ate fried food daily inside the home.

The associations between intake of foods inside and outside the home and risk of MI are shown in Table 3.

**Table 3:** Frequency of fried food intake and risk of nonfatal acute myocardial infarction in the Costa Rica Heart Study.

Frequency of eating fried foods	<1 time/week	1–3 times /week	4–6 times /week	daily	P for trend
Inside home					
Model 1^1^	1.0	0.82(0.68, 0.98)	0.86(0.67, 1.10)	1.19(0.97, 1.46)	0.0002
Model 2^2^	1.0	0.86(0.72, 1.06)	0.95(0.72, 1.25)	1.25(0.99, 1.57)	0.0005
Model 3^3^					
+ Fiber	1.0	0.86(0.70, 1.06)	0.94(0.71, 1.25)	1.20(0.95, 1.52)	0.002
+ Saturated fatty acid	1.0	0.83(0.67, 1.03)	0.88(0.67, 1.17)	1.09(0.86, 1.39)	0.03
+ Total energy intake	1.0	0.83(0.67, 1.02)	0.85(0.64, 1.13)	1.01(0.79, 1.28)	0.23
Model 4^4^	1.0	0.81(0.65, 1.00)	0.81(0.61, 1.09)	0.93(0.72, 1.19)	0.65
Outside of the home					
Model 1^1^	1.0	1.09(0.94, 1.26)	1.63(1.11, 2.37)	2.20(1.59, 3.04)	< .0001
Model 2^2^	1.0	1.17(0.99, 1.37)	1.74(1.14, 2.66)	2.18(1.51, 3.13)	< .0001
Model 3^3^					
+ Fiber	1.0	1.15(0.98, 1.36)	1.64(1.07, 2.52)	2.08(1.44, 2.99)	<0.0001
+ Saturated fatty acid	1.0	1.09(0.93, 1.29)	1.52(0.99, 2.34)	1.86(1.28, 2.70)	0.0003
+ Total energy intake	1.0	1.05(0.89, 1.25)	1.37(0.89, 2.12)	1.73(1.19, 2.51)	0.003
Model 4^4^	1.0	1.02(0.86, 1.21)	1.26(0.81, 1.95)	1.55(1.08, 2.30)	0.02

Odds Ratio (OR) of MI (95%CI) by frequency of fried food intake outside; All models used a fixed sample size of 2,154 case-control pairs

^1^ Model: ORs conditioned on matching variables (age, sex and area of residence).

^2^ Model 2: Adjusted for history of diabetes (yes/no), hypertension (yes/no), smoking (never, past, <10 cigarettes/d, 10–19 cigarettes/d, and ≥20 cigarettes/d), waist circumference (quintiles), physical activity (quintiles), income (quintiles), educational years, intake of alcohol (never, past, and tertiles of current drinkers) and occupation (retired, agriculture, plumbers, semi-skilled or driver, managers and administrators, professionals and others)

^3^ Model 3: Model 2 plus saturated fatty acid, fiber or total energy intake individually (all in quintile)

^4^ Model 4: Model 2 plus saturated fatty acid, fiber and total energy intake all together (all in quintile)

Compared to fried food intake <once per week at home (reference), daily intake of fried foods inside the home was associated with a 25% increase in the risk of MI (*P* for trend = 0.0005), whereas a 118% increase in risk was observed for those with daily intake of fried foods outside the home, after adjustment for non-dietary cardiovascular risk factors, including history of diabetes, history of hypertension, smoking, abdominal obesity, income, educational years, occupation, and alcohol intake. The association between daily intake of fried foods inside the home and risk of MI was attenuated and no longer significant after further adjustment for significant dietary confounders (OR 0.93, 95% CI 0.72, 1.19). However, the association between intake of fried foods outside of the home and risk of MI remained statistically significant (OR 1.55 95% CI 1.08, 2.30). The analysis using propensity score stratification yielded similar results (OR 1.57 95% CI 1.11, 2.21), which compared daily intake versus <1time/week of fried foods outside of the home. The OR for daily intake of fried foods versus <1time/week was 0.87 (0.69, 1.08) (*P* for trend = 0.96) for intake inside the home and 1.56 (1.11, 2.19) (*P* for trend = 0.02) for intake outside the home. Adjusting for type of cooking oil did not materially change the association between eating fried foods inside or outside of home and risk of MI. The OR for daily intake of fried foods versus <1time/week was 0.93 (0.72, 1.19) (*P* for trend = 0.71) for intake inside the home and 1.56 (1.06, 2.29) (*P* for trend = 0.02) for intake outside the home. In the sensitivity analysis of adjusting dietary patterns, the ORs comparing daily versus <1time/week intake of fried foods were 0.87 (95%CI: 0.68–1.11, *P* for trend = 0.98) for intake inside the home and 1.55 (95%CI: 1.06–2.26, *P* for trend = 0.02) for intake outside the home. The interactions between eating fried foods outside of the home and current smoking, physical activity, sex, and obesity on risk of MI were not significant (*P* for interaction >0.2 for all). The association between eating fried foods outside of the home and risk of MI was consistent in analyses stratified by sex, smoking, BMI, or physical activity (S1 Fig).

**Fig 1:**
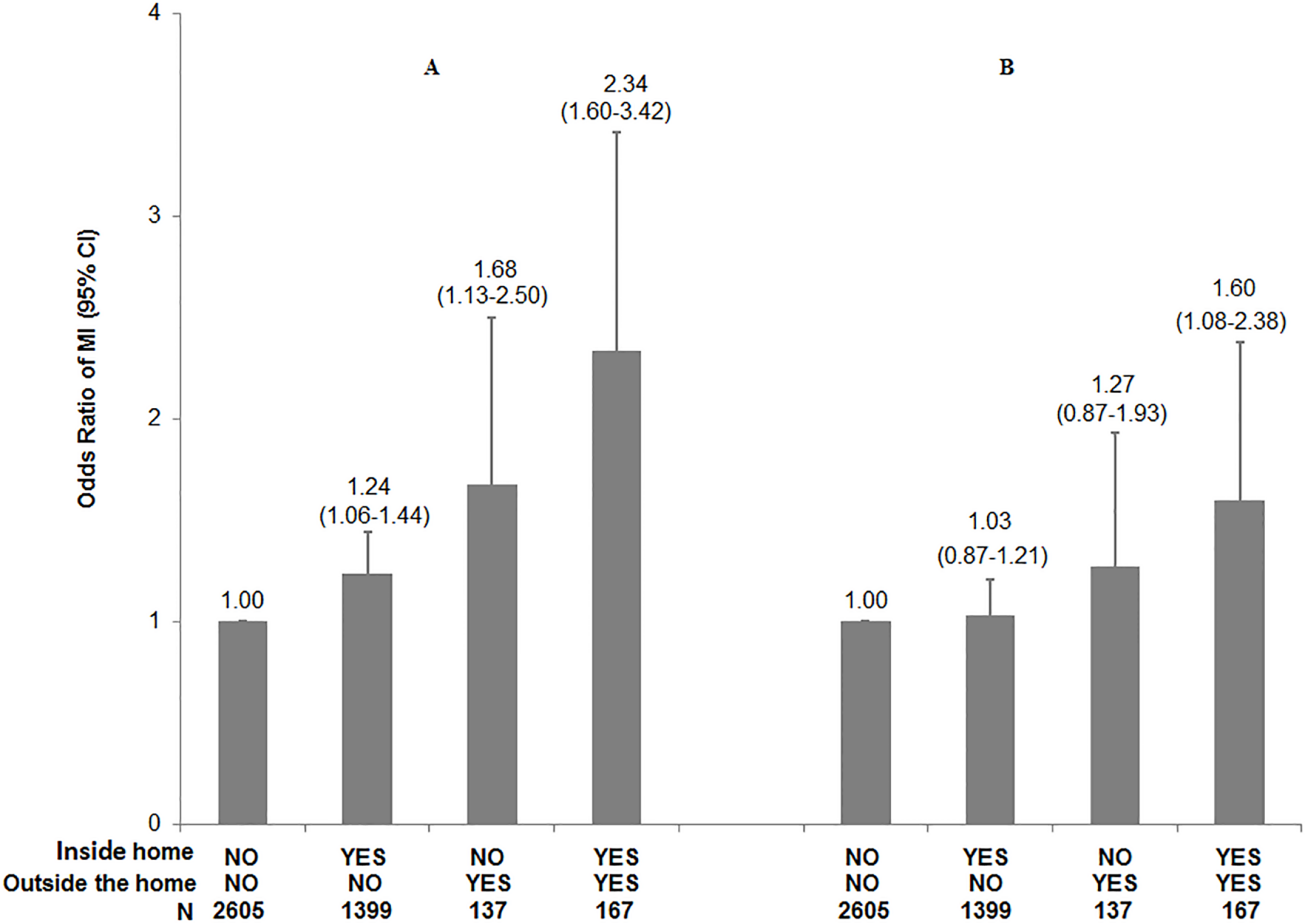
Joint effect of eating fried food ≥4 times/week at home and outside of the home. Odds Ratio (OR) of MI (95%CI) according to the joint category of fried food intake at home and outside of the home; All models used a fixed sample size of 2,154 case-control pairs, ORs conditioned on matching variables (age, sex and area of residence). A:, Adjusted for history of diabetes (yes/no), hypertension (yes/no), smoking (never, past, <10 cigarettes/d, 10–19 cigarettes/d, and ≥20 cigarettes/d), waist-hip-ratio (quintiles), physical activity (quintiles), income (quintiles), educational years, intake of alcohol (never, past, and tertile of current drinkers) and occupation (retired, agriculture, plumbers, semi-skilled or driver, managers and administrators, professionals and others); B: Further adjusted for saturated fat, fiber and total energy intake (all in quintile).

The independent and joint effects for fried food intake inside the home and outside of the home comparing intake ≥4 times/week to < 4 times/week are shown in Fig 1A (unadjusted for non-dietary confounders) and with additional adjustment for dietary factors in Fig 1B. Participants reporting fried food intake both inside the home and outside of the home ≥4 times/week had 60% higher risk of MI compared to those reporting intake < 4 times/week after adjustment for dietary and non-dietary confounders. A 27% increase in MI risk was found for those reporting only fried food intake outside of the home, whereas no association was found for those only reporting fried food intake inside the home.

## Discussion

In this case control study conducted in the Central Valley of Costa Rica, higher intake of total fried foods was associated with increased risk of MI. This association was mostly attributed to intake of fried foods outside the home. Daily intake of fried foods outside the home was associated with a 55% higher risk of MI, whereas no association was observed with daily intake of fried food inside the home after adjusting for smoking, physical activity, alcohol intake, education, occupation, income, waist circumference, fiber, saturated fat, energy intake, and history of diabetes or hypertension.

Total fried food intake has been associated with risk of heart disease in previous studies, a finding consistent with our current results [11,14] In the Nurses’ Health Study and the Health Professionals’ Follow-up Study [11] total fried food intake was associated with a 21% higher risk of heart disease. INTERHEART [14], a standardized case-control study of acute MI involving 5761 nonfatal MI cases and 10, 646 controls from 52 countries, observed a consistent positive association between acute MI and intake of fried foods. The OR comparing the highest with the lowest quartile of fried food intake was 1.13 (95%CI: 1.02–1.25, *P* for trend <0.0001) after multivariate adjustment. Intake of both fried foods inside and outside the home in our study was higher than those in previous studies (OR, 1.60, 95% CI 1.08, 2.38). Although the rationale for this result is uncertain, it is reasonable to hypothesize that the type of oil used for cooking could explain some of these differences. The use of palm oil and high trans soybean oil for cooking, previously associated with heart disease, is higher in Costa Rica than in other countries where studies have been conducted [25,26]. In contrast to other previous studies, no association between fried food intake and risk of heart disease was found in the Spanish Cohort of the European Investigation into Cancer and Nutrition where olive oil is the main type of fat used for cooking [15].

Consistent with our study, fried food intake away from home has been associated with risk of heart disease in previous studies. In the Nurses’ Health Study and the Health Professionals’ Follow-up Study [11], fried food intake away from home was associated with heart disease. The magnitude of the association in the Costa Rican Heart Study was greater than in the U.S cohort study (OR 1.18, 95% CI 0.99, 1.39). The frequency of fried food intake away from home was relatively low (< 10% reporting eating away from home >4 times per week) in our study. However, the 55% increase in risk of MI observed in our study is of concern given that it is likely that intake of foods outside of the home will continue to increase in middle-income countries [2,6].

Frying is a commonly used cooking technique in Costa Rican homes. In fact, over 85% of the studied population reported intake of fried foods inside the home at least once per week. The most commonly fried foods in Costa Rican homes include plantains, potatoes, cassava, maize turnovers, and rice. Interestingly, we did not find an association between intake of fried foods inside the home and risk of MI despite of the potential detrimental health effects of fried foods [26]. This null association could be explained in part by lower oxidation products in fried foods inside the home. Frying increases the amount of cholesterol and other lipid oxidation products mostly when cooking meat, fish, and vegetables, as opposed to carbohydrate rich foods [27,28]. Frying foods inside the home involves shorter frying times, lower temperatures, and pan-frying (versus deep frying). These characteristics of frying foods inside the home could have contributed to the observed result [29,30]. In contrast, commercially prepared fried foods are often more likely to re-use oil several times and deep-fry at higher temperatures [31,32].

Major strengths of our study were the ability to evaluate fried food inside and outside of the home in the context of a middle-income country with different dietary patterns and lifestyles than previous studies. Other strengths include the large sample size, high validity and participation (98% among cases and 88% among controls), restriction of recruitment to survivors of a first nonfatal MI in a pre-specified catchment area, and the use of randomly selected population-based controls. In addition, we collected detailed information on diet using the standardized FFQ designed and validated specifically for the studied population. Furthermore, our study focused on eating patterns rather than specific nutrients, which translates more easily to health recommendations and public understanding.

Several limitations also warrant consideration. First, the experience of having a MI could have modified cases responses. To minimize possible recall bias, dietary data collection was conducted in the subjects’ home as close as possible to hospital discharge (26±10 days after the MI). For most cases (81%), data collection was completed within <14 days [1,18,33]. Given the observational nature of our study, we cannot prove causality. Similarly to other observational studies, it is difficult to rule out residual confounding, despite careful control for potential confounders in the analyses. Future prospective studies and clinical trials in this area are warranted. Additionally, we did not take into account the specific fried foods our participants ate, nor the duration, temperature, or methods in which they were cooked. Nonetheless, our results might underestimate the true magnitude of the effect because of measurement error and over-adjustment for covariates that might be on the causal pathway, such as hypertension, diabetes or obesity.

In sum, we found that frequent consumption of fried foods outside of the home is associated with a higher risk of MI. This finding suggests that the trend towards higher consumption of commercially available foods outside the home could have detrimental effects on the risk of cardiovascular disease in middle-income countries.

## Supporting information

S1 Fig Multivariate-adjusted odds ratio of MI stratified by potential confounders1,2. 1 Odds ratio of MI comparing eating fried foods outside daily versus less than once per week by conditional logistic regression adjusted for history of diabetes (yes/no), hypertension (yes/no), smoking (never, past, <10 cigarettes/d, 10–19 cigarettes/d, and ≥20 cigarettes/d), waist-hip-ratio (quintiles), physical activity (quintiles), income (quintiles), educational years, intake of alcohol (never, past, and tertiles of current drinkers), and occupation (retired, agriculture, plumbers, semi-skilled or driver, managers and administrators, professionals and others), besides the stratification factor.
2 unconditional logistic regression adjusted variables listed above and age, sex and area of residence, besides the stratification factor.
(TIF)
