# Supplementary figures and images for: Fried food intake and risk of nonfatal acute myocardial infarction in the Costa Rica Heart Study
Source: PLoS One. 2018 Feb 15;13(2):e0192960. doi: 10.1371/journal.pone.0192960 (PMC5813981; doi:10.1371/journal.pone.0192960)

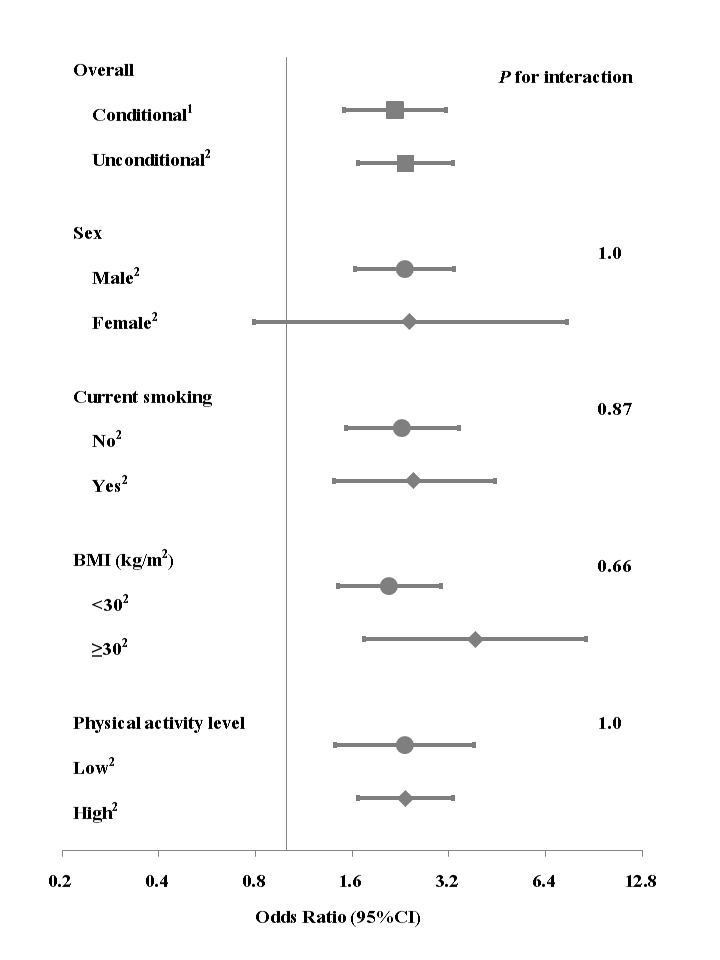

Supplement: S1 Fig — 1 Odds ratio of MI comparing eating fried foods outside daily versus less than once per week by conditional logistic regression adjusted for history of diabetes (yes/no), hypertension (yes/no), smoking (never, past, <10 cigarettes/d, 10–19 cigarettes/d, and ≥20 cigarettes/d), waist-hip-ratio (quintiles), physical activity (quintiles), income (quintiles), educational years, intake of alcohol (never, past, and tertiles of current drinkers), and occupation (retired, agriculture, plumbers, semi-skilled or driver, managers and administrators, professionals and others), besides the stratification factor. 2 unconditional logistic regression adjusted variables listed above and age, sex and area of residence, besides the stratification factor. (TIF) [file pone.0192960.s001.tif]
